# Comparison of Two Main Orthokeratology Lens Designs in Efficacy and Safety for Myopia Control

**DOI:** 10.3389/fmed.2022.798314

**Published:** 2022-04-01

**Authors:** Weiwei Lu, Rui Ning, Kai Diao, Yang Ding, Ruru Chen, Lei Zhou, Yan Lian, Colm McAlinden, Francis W. B. Sanders, Fangfang Xia, Jinhai Huang, Wanqing Jin

**Affiliations:** ^1^Eye Hospital and School of Optometry and Ophthalmology, Wenzhou Medical University, Wenzhou, China; ^2^State Key Laboratory of Optometry, Ophthalmology, and Vision Science, Wenzhou, China; ^3^Department of Ophthalmology, Ningbo Eye Hospital, Ningbo, China; ^4^Department of Ophthalmology, Singleton Hospital, Swansea Bay University Health Board, Singleton, United Kingdom; ^5^Department of Ophthalmology, Eye Institute, Eye and ENT Hospital, Fudan University, Shanghai, China; ^6^NHC Key Laboratory of Myopia (Fudan University), Key Laboratory of Myopia, Chinese Academy of Medical Sciences, Shanghai, China; ^7^Shanghai Research Center of Ophthalmology and Optometry, Shanghai, China

**Keywords:** orthokeratology, myopia control, safety, efficacy, design

## Abstract

**Purpose:**

This study aimed to compare the efficacy and safety of corneal refractive therapy (CRT) lenses and vision shaping treatment (VST) lenses for myopia control in children.

**Methods:**

Medical records of 1,001 children (2,002 eyes) who had been fitted with orthokeratology lenses for over 1.5 years were retrospectively reviewed. We collected the clinical data of four types of orthokeratology (OK) lenses available: one CRT lens (brand: CRT) and three VST lenses (brands: Euclid, Alpha, and Hiline) over 1.5 years. Results were compared and analyzed using a one-way ANOVA and Pearson's chi-square test.

**Results:**

Axial length elongation in the CRT lens group was 0.13 ± 0.02 mm faster than that in the Euclid lens, 0.1 ± 0.02 mm faster in the Alpha lens, and 0.08 ± 0.02 mm faster in the Hiline lens over the 1.5-year period (all *P* < 0.05). Among the subjects, 37.3% of them using the CRT lens experienced more than 1 D of refractive growth, compared with 20.2–30.8% of subjects wearing the three groups of VST lenses (all *P* < 0.05). A lower incidence of total adverse events was found with the CRT lenses compared with the VST lenses (*P* < 0.05), especially corneal staining. No difference was found in axial length elongation, refraction growth, and incidence of adverse events among the three types of VST lenses (all *P* > 0.05).

**Conclusions:**

Compared with the VST lenses, CRT lenses demonstrated a weaker effect on myopia control but with a better safety profile. Different types of VST lenses had similar efficacy and safety in the context of controlling myopia progression.

## Introduction

It is predicted that, by 2050, 49.8% of the global population (4,758 million people) will have myopia and 938 million people will have high myopia (1). Therefore, myopia has become one of the leading causes of visual impairment worldwide (2), requiring the development of more varied management strategies to provide for the breadth of clinical presentations. Numerous earlier studies and meta-analyses spanning over 30 years have demonstrated that orthokeratology (OK) is an effective means of controlling myopia and slowing axial length (AL) growth (3–6). Most researchers hold an opinion that the mid-peripheral steepening of the cornea by overnight OK lens leads to the induction of peripheral myopic defocus and thus may reduce the visual feedback for axial elongation, leading to slower myopic progression (7, 8).

There are many different OK lens brands available in the market, which can make it challenging to choose the most appropriate lens for myopic children. In order to help patients to obtain the best myopia control, it is important to understand whether there is any difference in the efficacy of myopia control between the different available designs of OK lenses. Previous studies found a similar relative peripheral refraction changes between two OK lenses (9) and a similar efficacy on slowing AL elongation between four different brands of OK lenses with the same lens design (10). However, studies in recent years have demonstrated that a smaller center treatment-zone diameter in topographic tangential maps could lead to a greater effect on slowing AL elongation (11). Pauné et al. (12) also observed that efficacy in the context of controlling myopia progression was improved by modifying the design of the OK lens [reducing the optic zone diameter (OZD)]. However, whether the efficacy in myopic control differs between the available designs of OK lenses remains unclear.

Multiple systematic reviews have confirmed that there is sufficient evidence that OK is a safe option for the correction of myopia (7, 13–17). However, there are still some differences in the incidence of significant adverse events between the various studies. Peike et al. (16) and Takahiro et al. (17) reported 6.9% and 11.4% of adverse events, respectively. Thus, it would be valuable for patients to compare the safety of different OK lens designs. However, to our knowledge, there is no published study comparing the incidence of adverse events between different OK lens designs.

To better understand myopia control and answer the question of whether the effectiveness and safety of OK lenses differ between different designs, we collected and analyzed clinical data in refractive error, AL, and ocular surface condition between four kinds of OK lenses over a 1.5-year period. The current study aimed to compare the efficacy and safety of the two main OK lens designs in clinics: corneal refractive therapy (CRT) and vision shaping treatment (VST).

## Materials and Methods

### Participants

The clinical records of 1,001 children (aged 8–15 years old) were collected and analyzed. The subjects attended the Eye Hospital of Wenzhou Medical University, Zhejiang Eye Hospital, or Ningbo Eye Hospital between June 2017 and January 2019. The involved subjects had been fitted with OK lenses for over 1.5 years. The initial spherical equivalent refraction ranged from −6.5 to −0.5 D. All subjects provided written informed consent following an in-depth discussion of the possible risks and nature of wearing an OK lens. The study was approved by the local ethics committee and was performed in accordance with the guidelines of the Declaration of Helsinki. All subjects were screened to evaluate if they met the inclusion and the exclusion criteria. The inclusion criteria were the following: (1) age of 8 to 15 years old, as this is the age range within which the fastest rates of myopia progression have been observed (18); (2) the lens brand including CRT or VST (Euclid, Alpha, or Hiline); (3) the time of OK wear of more than 14 months; (4) the monocular corrected distance visual acuity (LogMAR) equal to or less than 0; (5) normal anterior segment, fundus, and other ocular structures after initial examinations; (6) intraocular pressure < 21 mmHg; (7) no use of atropine treatment before and during OK use; and (8) discontinued OK wear for <1 month during the 1.5 years. The exclusion criteria were the following: (1) history of atropine treatment, other defocus spectacles, or other contact lenses; (2) eye diseases except for refractive error; (3) wore other types of OK lenses or changed the brand of the lens; and 4) more than 0.25 D change in alignment curve (AC) in VST design between the patient's first OK lens and their second OK lens or more than 25 um change in reverse zone (RZD) for CRT lenses.

### Materials

In this retrospective study, we collected the clinical data of two main types of OK lenses available, namely CRT and VST lenses. According to the manufacturer's documentation, the CRT lens design was divided into only three curves, including the Paragon CRT (Paragon Vision Sciences, Inc., USA). In this study, the VST lens was defined as an OK lens with four or more curves, including three OK brands: Euclid (Euclid Systems Corporation, Herndon, Virginia, USA), Alpha (Alpha Corporation, Japan), and Hiline (Nanpeng Hiline Inc., Taiwan, China) lenses. The VST lens design is based on corneal curves, so the end of a peripheral curve touches the edge of the cornea. The peripheral curve in the VST lens is the edge of the lens and is aspheric in design. The CRT lens is designed to have congruent anterior and posterior surfaces, each consisting of the following three zones: central spherical zone, a mathematically designed sigmoidal corneal proximity “return zone,” and a non-curving “landing zone.” The lens design also includes a convex elliptical edge terminus smoothly joining the anterior and posterior surfaces (19). The edge of the lens does not touch the cornea, which may increase tear exchange. The compression factor is the extra power included in OK lenses, excluding the manifest refractive correction of individual patients, which are added on to the target of OK to allow for a gradual change in refraction over the course of the day after removal of the OK lenses. The compression factor of all three VST lenses was +0.75 D, and the compression factor of CRT lenses was +0.5 D. A summary of the four lenses' characteristics and standard parameters that were used is described in Table 1, based on details provided in the manufacturer's fitting guide.

**Table 1:** Characteristics and standard parameters of four different orthokeratology (OK) lenses.

**Lenses**	**Design**	**Curves**	**Material**	**Dk (×10^**−11**^)**	**CT**	**DK/t**	**WA**	**CF**	**OZD**	**RCW**	**ACW**	**PCW**	**TD**
CRT	CRT	3	HDS 100	100	0.16	62.5	42°	+0.50	6.0	1.0	1.25	(LZW)	10.5
Euclid	VST	5	Boston Equalens II	127	0.22	57.7	30°	+0.75	6.2	0.5	1.2	0.5	10.6
Alpha	VST	5	Boston EM	104	0.22	47.3	35°	+0.75	6.0	0.6	1.3	0.4	10.6
Hiline	VST	5	Boston Equalens II	127	0.26	48.8	30°	+0.75	6.0	0.5	1.3	0.5	10.6

*Dk, oxygen permeability (10^-11^ cm^2^/s) (ml O_2_/ml mmHg); CT, central thickness (mm); WA, wetting angle; CF, compression factor (D); OZD, optic zone diameter (mm); RCW, reverse curve width (mm); ACW, alignment curve width (mm); PCW, peripheral curve width (mm); LZW, landing zone width; TD, total diameter*.

### Clinical Data Collection

All subjects underwent a standardized anterior segment examination before wearing OK lenses: a slit lamp examination including tear film break-up time and meibomian gland function, corneal topography, ocular AL, specular microscopy, and subjective refraction. Subjects were followed-up after initiation of OK wear according to a standardized visit schedule at 1 day, 1 week, 1 month, 3 months, 6 months, and at 3 monthly intervals thereafter. The general ocular health, lens fitting, visual acuity, and AL were collected at every visit. AL was measured five times with an Intraocular Lens Master (IOL-Master 500, Zeiss, Germany) at every 3-month visit.

Clinically, the patient wore a trial lens that was identical to the final best-fitting lens identified during the initial lens fitting and then it was over-refracted to produce their therapeutic OK lens. The amount of refractive change that occurred over this period was equal to the difference between the initial and the final (after ~1.5 years) over-refraction values. If the amount of refraction growth was less than or equal to 0 D, it was defined as “no myopic progression.” It was considered as “slow myopic progression” when the amount of refraction growth was between 0 and 1 D. Finally, it was considered “fast myopic progression” if the amount of refraction growth was more than 1 D. As for adverse events, the database of all the Hiline (225 subjects) and a small proportion of CRT (59 subjects) lenses were collected from the Ningbo Eye Hospital, of which medical records regarding eye health condition was incomplete and was not kept up with the other centers in this study. Therefore, we excluded the data on ocular safety collected from this eye hospital and reanalyzed the remainder data on ocular safety collected from the other hospitals (204 subjects in the CRT OK group, 277 subjects in the Euclid OK group, and 236 subjects in the Alpha OK group). Adverse events included corneal staining, conjunctival inflammation, and eyelid gland abnormality, mainly caused by unskilled OK wearing practice, atypical eyelid or corneal morphology, poor tear film quality, and inadequate lens care. Corneal staining was recorded according to the Cornea and Contact Lens Research Unit (CCLRU) grading scale (20).

### Statistical Analysis

All statistical analyses were performed using SPSS software (version 25; IBM Corporation, Armonk, NY). The measured data were tested using the Kolmogorov-Smirnov test, and results with a normal distribution were presented as the mean ± *SD*. Gender, myopic progression, and ocular safety were presented as percentages of subjects. Snellen acuity was converted to LogMAR for analysis. The visual acuity was non-normally distributed and was represented as median (p. 25, p. 7 5). To avoid statistical issues with the use of both eyes from the same subject (21), only the data of right eyes were used in the statistical analyses. With regard to ocular safety, data for both eyes were included and analyzed. The incidence of adverse events was calculated as a percentage of eyes. Recurrences of the same adverse event in the same or fellow eye at any of the subsequent study visits were classified as separate events, and bilateral events were counted as two separate events. Visual acuity was analyzed with the Kruskal-Wallis H non-parametric tests, and the Nemenyi test was used for multiple pairwise comparisons. Age, spherical equivalent refractive errors (SER), AL, total follow-up times, and 1.5-year change in AL were compared among the four groups using independent sample one-way ANOVA analyses. Fisher's least significant difference (LSD) method was used to correct for multiple comparisons. The differences in gender, myopic progression, and ocular safety among groups were tested using Pearson's chi-square test for multiple sample rates with the Bonferroni method of correction for multiple comparisons. A *P* < 0.05 was considered statistically significant.

## Results

The mean age was 10.45 ± 1.79 years old, and the initial mean spherical equivalent refraction was −3.26 ± 1.34 D. There were 263 subjects in the CRT OK group, 277 subjects in the Euclid OK group, 236 subjects in the Alpha OK group, and 225 subjects in the Hiline OK group. There was no statistically significant difference at baseline for sex among the four groups (Table 2). Baseline age, initial spherical equivalent, initial AL, and initial corneal topography parameters were compared among the four OK lenses, and no statistically significant difference was found in any baseline data (all *P* > 0.05). There was also no significant difference in the total period of OK lenses among the four groups. There was no significant difference in data between right eyes and left eyes (all *P* > 0.05).

**Table 2:** Baseline demographics for the four groups.

	**CRT**	**VST**			***P***
		**Euclid**	**Alpha**	**Hiline**	
Numbers	263	277	236	225	–
Male, %	48.29	45.68	48.31	48.07	0.913
Age (Y) (95%CI)	10.52 ± 1.85	10.31 ± 1.65	10.67 ± 1.81	10.32 ± 1.83	0.079
	(10.29–10.75)	(10.12–10.51)	(10.43–10.89)	(10.07–10.56)	
SER (D) (95%CI)	−3.22 ± 1.42	−3.14 ± 1.02	−3.45 ± 1.41	−3.25 ± 1.52	0.060
	(−3.39 to −3.05)	(−3.25 to −3.01)	(−3.63 to −3.27)	(−3.45 to −3.05)	
AL (mm) (95%CI)	24.91 ± 0.82	24.97 ± 0.81	25.05 ± 0.84	24.87 ± 0.86	0.111
	(24.81–25.01)	(24.88–25.07)	(24.94–25.16)	(24.76–24.98)	
Follow-up times (M) (95%CI)	18.79 ± 2.13	18.64 ± 1.88	18.64 ± 2.23	19.06 ± 1.85	0.078
	(18.54–19.05)	(18.41–18.86)	(18.36–18.93)	(18.82–19.30)	

*SER, spherical equivalent refraction; D, diopters; AL, axial length*.

### Axial Length Elongation

**Figure 1:**
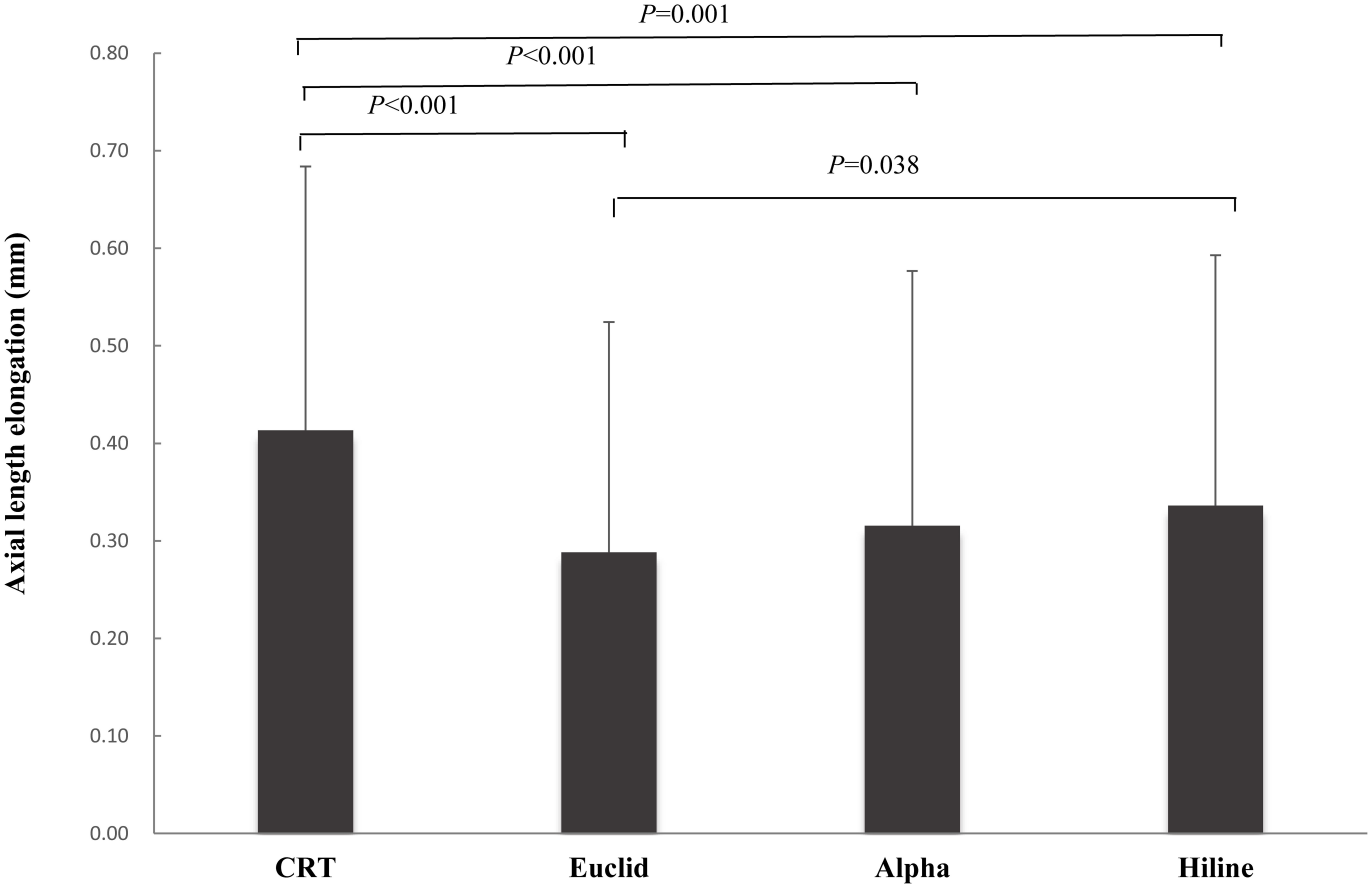
Comparison of axial length elongation after one and a half years of orthokeratology (OK) lens wear between four different lens types.

After the OK lenses had been worn for 1.5 years, the increase in AL were 0.41 ± 0.27 mm, 0.29 ±0.24 mm, 0.32 ± 0.26 mm, and 0.34 ± 0.25 mm in the CRT, Euclid, Alpha, and Hiline groups, respectively (Figure 1). AL elongation in the CRT group was 0.13 ± 0.02 mm greater than that in the Euclid groups (*P* < 0.001), 0.1 ± 0.02 mm was greater than that in the Alpha group (*P* < 0.001), and 0.08 ± 0.02 mm was greater than that in the Hiline group (*P* = 0.001). The AL elongation in the Hiline group was 0.05 ± 0.02 mm greater than that in the Euclid group (*P* = 0.037). There was no significant difference between the Alpha group and the Euclid group (*P* = 0.249) or between the Hiline group and the Alpha group (*P* = 0.358).

### Rates of Myopic Progression

Corneal refractive therapy lenses demonstrated the effective control of myopic progression (no myopic progression, refraction growth ≤ 0 D) in 26.7% of subjects, which was calculated from the change in over-refraction with the same trial lens. Similarly, 22.1–32.2% of subjects with VST lenses (Table 3) also demonstrated no significant change in refraction. There was no significant difference in the percentage of no myopic progression among the four groups (*P* = 0.285). However, slow myopic progression (refraction growth ranging from 0 to 1 D) was observed in 36% of subjects with CRT lenses and 37–56.6% of subjects with VST lenses (Table 3). Slow myopic progression was observed in the CRT group, showing a statistically significant lower incidence than that found in the Euclid and Alpha groups (*P* < 0.001). Meanwhile, we found that 37.3% of subjects using CRT lenses demonstrated fast myopic progression (refraction growth ≥ 1 D), which had a significantly higher incidence in fast myopic progression in comparison to 20.2–30.8% of subjects in the other three groups (Table 3, all *P* < 0.05). The corresponding trend in fast myopic progression was also observed in AL elongation with significant differences observed between CRT lenses and the VST lenses (*P* < 0.001) (Table 3). No myopic progression in terms of AL elongation was observed at a significantly lower incidence with CRT lens wear compared to that found with any VST lens (*P* < 0.001).

**Table 3:** Comparison of rates of myopic progression among four kinds of OK lenses over a 1.5-year period of use.

	**No progression**	**Slow progression**	**Fast progression**
	**ER: ≤ 0.00 D**	**ER: 0.00–1.00 D**	**ER ≥ 1.00 D**
	**AL: ≤ 0.15 mm**	**AL: 0.15–0.4 mm**	**AL > 0.4 mm**
**Refraction growth**			
CRT	26.7%	36.0%^*^^,^^†^	37.3%^*^^,^^†^^,^^‡^
Euclid	22.1%	56.6%^*^	21.3%^*^
Alpha	26.3%	53.5%^†^	20.2%^†^
Hiline	32.2%	37.0%	30.8%^‡^
**Axial length elongation**			
CRT	14.8%^*^^,^^†^^,^^‡^	33.1%	52.1%^*^^,^^†^^,^^‡^
Euclid	29.5%^*^	40.0%	30.5%^*^
Alpha	27.1%^†^	36.0%	36.9%^†^
Hiline	25.4%^‡^	37.5%	37.1%^‡^

*Corrected pairwise comparison of P-values are represented by*

* *, significant (P < 0.05) difference between CRT and Euclid*.

† *Significant difference between CRT and Alpha*.

‡ *Significant difference between CRT and Hiline. Pairwise comparison is corrected for multiple comparisons. ER, estimated refraction. No progression means reduction (or no change) in refraction or less than (equal to) 0.15 mm AL elongation. Slow progression means 0 to 1 D refraction growth or 0.15 to 0.4 mm AL elongation. Fast progression means more than 1 D refraction growth or more than 0.4 mm AL elongation*.

### Visual Acuity

The visual acuity (LogMAR) after OK lenses had been worn for 1.5 years was 0 (0, 0.1) in the CRT group, 0 (0, 0) in the Euclid group, 0 (0, 0.1) in the Alpha group, and 0.1 (0, 0.1) in the Hiline group. We only found that the Hiline group had a slightly worse visual acuity than the other three groups (*P* < 0.001). The CRT, Euclid, and Alpha groups had similar visual acuity after wearing OK lenses for 1.5 years (all *P* >0.05).

### Ocular Safety

The average incidence of total adverse events was observed at 13.3% for all OK lenses. There was a statistically significant (*P* < 0.001) lower incidence of total adverse events in the CRT group at 10.3% (54 eyes), in comparison to 15.1% (84 eyes) with Euclid lens and 14.4% (68 eyes) with Alpha lens (Table 4). Corneal staining was the most frequent adverse event found in all groups. In the CRT group, 5.9% had corneal staining, showing a statistically significant higher incidence than that found in the Euclid and Alpha groups (*P* < 0.001). There was a lower incidence of moderate-to-severe corneal staining in the CRT group in comparison to that found in the Euclid group (*P* = 0.033). Otherwise, no significant difference in conjunctival inflammation and meibomian gland abnormality was found between the three groups (all *P* > 0.05).

**Table 4:** Comparison of the incidence of total adverse events and specified adverse events over a 1.5-year period of different OK lens wear.

**OK lenses**	**Total adverse events**	**Corneal staining**	**Conjunctival inflammation**	**Eyelid gland abnormality**
CRT	10.3% (54)^*^^,^^†^	5.9% (31)^*^	4.0% (21)	0.4% (2)
Euclid	15.1% (84)^*^	10.3% (57)^*^	3.2% (18)	1.6% (9)
Alpha	14.4% (68)^†^	8.5% (40)	5.5% (26)	0.4% (2)
*P*-value	< 0.001	0.033	0.187	0.052

*Corrected pairwise comparison of P-values are represented by*

* *, significant (P <0.05) difference between CRT and Euclid, and*

† *, significant difference between CRT and Alpha. Pairwise comparison is corrected for multiple comparisons. Recurrences of the same adverse event in the same or fellow eye at any of the subsequent study visits were classified as separate events, and bilateral events were counted as two separate events*.

## Discussion

With the growing popularity of OK lens use, an increasing amount of attention has been paid to the efficacy of OK lenses in controlling myopia. At the same time, there have been an increasing number of OK lenses available, incorporating varying lens designs and different materials. This has led to a discussion as to whether these different designs might lead to differences in efficacy and ocular safety. In this study, the efficacy and safety were both evaluated for the two main types of OK lenses (CRT and VST) by collecting and analyzing the clinical data of 1,001 myopic schoolchildren over a 1.5-year period.

Three VST lenses (Figure 1) exhibited very similar efficacy in slowing AL elongation in the context of myopia, except a 0.05 mm difference between the Euclid and Hiline lenses. Numerous previous studies relating to efficacy mainly involved the VST lens design. Chen et al. (10) collected data on four brands of OK lenses, all belonging to the VST design, which are entirely different from those in the current study, and reported a similar AL elongation for 1.5 years, ranging from 0.24 to 0.32 mm. These AL elongations aligned well with the results observed in the current study for the VST lenses. Cho et al. (22) reported a 0.3 mm increase in AL with another brand of VST lens, which is also within the range of 0.29 to 0.34 mm found in VST lenses studied herein. These results and our findings indicated that most brands of VST lenses had a very similar efficacy on myopia control. However, no study until now has compared the efficacy with two main designs of OK lenses (CRT and VST). In this study, Figure 1 shows that the AL elongation was 0.41 mm with the CRT lens and 0.29 to 0.34 mm with the three VST lenses. This highlights the surprising result that AL elongation with the CRT lens was significantly faster than with the VST lenses.

The efficacy of myopia control was evaluated not only by the AL elongation but also by the other crucial parameters. In this study, a weaker effect on controlling myopia in the CRT lens was also observed in terms of refraction growth. The refraction growth (Table 3) showed that 37.3% of the subjects wearing CRT lenses had “fast myopic progression” (refraction growth was more than 1 D), which was significantly higher than the incidence (20.2–30.8%) in any of the VST OK lenses. A significantly lower incidence of “slow myopia progression” was also found in the CRT lens group compared to all of the VST lens groups. This raises the question of why those using the CRT lens demonstrated a faster rate of myopia progression than those using VST lenses. Recently, some studies (11, 12, 23, 24) have focused on OK lens design and have found that the design of OK lenses may have a significant influence on the efficacy of the OK lens on slowing myopia progression. Some important design parameters highlighted include center treatment-zone diameter, compression factor, and the number of curves incorporated into an OK lens. The center treatment-zone diameter may be an important influencing factor for the efficacy of the OK lenses. Pauné et al. (12) and Yang et al. (11) found a smaller center treatment-zone diameter led to a greater effect on slowing AL elongation. The authors proposed that the smaller center treatment-zone diameter caused more rays to go through the steepened mid-peripheral defocusing ring, which caused the retina to receive more peripheral myopic defocus, resulting in the better efficacy of the OK lens in terms of slowing axial growth. In a previous study from 2018, Marcotte-Collard et al. (23) analyzed topographic tangential maps and demonstrated that CRT lenses generated a larger central treatment-zone diameter than VST lenses both horizontally and vertically. These previous results strongly support the conclusion observed in this current study. Furthermore, the efficacy of OK lenses may be related to the compression factor. Wan et al. (24) found that AL elongation with OK lenses with an increased compression factor (1.75 D) was slower than those with conventional compression factor (0.75 D) during the first month of lens wear. To our knowledge, only the CRT lens had a lower compression factor (0.5 D) than the three VST lenses (0.75 D). As a consequence of the outcomes highlighted above, we also suspect that the lower compression factor in the CRT lenses may be a factor underlying the observed lower efficacy of the CRT lens. However, further investigation is needed to explore the long-term effects of increased compression factor on myopia control. Third, the number of curves may be another influencing factor for lower efficacy with the CRT lens. The CRT lens was designed with three curves, whereas the VST lenses were designed with five or more curves. However, this possible relationship needs further investigation. Besides the design of the OK lenses, the material may also be a possible influencing factor for different efficacy observed between lenses. The center thickness was thinnest in the CRT lens and the rigidity of the CRT lens was the least among the four OK lenses studied, which may lead to less mechanical action on the cornea, resulting in faster myopia progression compared to the VST lenses.

Safety is another important factor to be considered when OK lenses are used in myopic children. There was already a good body of evidence to support the safety of OK lenses in general (6, 13–17, 25). The average incidence of total adverse events was 13.3% in the current study. Li et al. (25) summarized the results of 9 well-conducted studies and found a similar incidence of adverse events-−13.8% (ranging from 3.8 to 29%)—aligning with the observed range of 10.3–15.1% for the current study. In this study, the results showed that the CRT lens had a statistically significant advantage in ocular safety compared with VST lenses, particularly with regard to corneal staining. We speculate that the material used is likely to be a key reason behind this finding, especially the oxygen transmissibility (DK/t) of the material. CRT lenses are the thinnest and have the highest DK/t among all lenses, which may allow more oxygen to reach the cornea (26) and hence reduce corneal adverse events. According to the manufacturers' documentation, compared with VST lenses, whose aspheric end design touches the edge of the cornea, the tangent design at the edge of the CRT lens increases the exchange of the tear film to the cornea underneath the lens and mobility of the lens, which may be part of the reason for less corneal staining seen with this lens. The CRT lens may be a better myopia management option when considering long-term ocular safety for children who are more susceptible to adverse events or those with worse tear film quality.

This study with a large sample size revealed that different designs of OK lenses could be associated with a difference in the efficacy of myopia control. Personalized OK lens design may be important to improve the efficacy of myopic control in the clinical settings. Interestingly, a new CRT lens design with a smaller central treatment-zone diameter (5 mm) is being produced, aimed at improving the efficacy of myopia control. The efficacy of this new CRT lens design needs to be further evaluated with clinical studies in the future. Furthermore, there are many confounding variables affecting the efficacy of OK lenses, including genetic background, living environment or habits, school vacation periods, and seasons. In this study, some of these variables (age, sex, refraction, AL, and corneal topography parameters) had been included in the statistical analysis. Meanwhile, a large sample size (the clinical data of 1,001 myopic schoolchildren) and the long time period of follow-up (a 1.5-year period) in the current study may reduce the effect of some of the other confounding variables on results and conclusions. Further prospective intervention studies are required with the adequate matching of baseline characteristics between treatment groups to allow for a more rigorous interrogation of the efficacy of OK lens design on myopia progression independent of confounding variables. Another point to consider in future studies would be to clarify why different lens designs achieve different efficacies in myopia control. This could include observing the differences in corneal topography (such as the diameter of the central treatment area and decentration during treatment), observing the distribution of refracted light on the peripheral retinal, and how these relate to factors that may evoke more or less efficacy in myopia control.

In summary, this study revealed that CRT lenses exhibited excellent ocular safety, but with worse myopia control compared with VST lens design. Personalized OK lens design may be a significant factor in improving the efficacy of myopia control. Future studies could include the corneal topographical analysis and the peripheral retinal defocus analysis of the different lens designs to guide personalized OK lens design and clinical fitting.

## Data Availability Statement

The original contributions presented in the study are included in the article/supplementary material, further inquiries can be directed to the corresponding author/s.

## Ethics Statement

The studies involving human participants were reviewed and approved by Eye Hospital and School of Optometry and Ophthalmology, Wenzhou Medical University, Zhejiang. Written informed consent to participate in this study was provided by the participants' legal guardian/next of kin.

## Author Contributions

WL and WJ designed this study. WL, WJ, YD, LZ, FX, and KD collected and measured data. RC, YL, RN, and CM analyzed data. WL, WJ, CM, FS, and JH wrote this manuscript. All authors made the final approval of this manuscript.

## Funding

This study was supported by the Wenzhou Science and Technology Bureau Basic Project (Grant No. Y20210203, Zhejiang, China), the EYE and ENT Hospital of Fudan University High-level Talents Program (Grant No. 2021318, Shanghai, China), and the Innovation and Guidance project of Eye Hospital affiliated to Wenzhou Medical University (YNCX3201907, Zhejiang, China). The sponsor or funding organization had no role in the design or conduct of this research.

## Conflict of Interest

The authors declare that the research was conducted in the absence of any commercial or financial relationships that could be construed as a potential conflict of interest.

## Publisher's Note

All claims expressed in this article are solely those of the authors and do not necessarily represent those of their affiliated organizations, or those of the publisher, the editors and the reviewers. Any product that may be evaluated in this article, or claim that may be made by its manufacturer, is not guaranteed or endorsed by the publisher.
